# Oridonin induces apoptosis and cell cycle arrest of gallbladder cancer cells via the mitochondrial pathway

**DOI:** 10.1186/1471-2407-14-217

**Published:** 2014-03-21

**Authors:** Runfa Bao, Yijun Shu, Xiangsong Wu, Hao Weng, Qian Ding, Yang Cao, Maolan Li, Jiasheng Mu, Wenguang Wu, Qichen Ding, Zhujun Tan, Tianyu Liu, Lin Jiang, Yunping Hu, Jianfeng Gu, Yingbin Liu

**Affiliations:** 1 Institute of Biliary Tract Disease, Shanghai Jiao Tong University School of Medicine, No. 1665 Kongjiang Road, Shanghai 200092, China; 2 Laboratory of General Surgery and Department of General Surgery, Xinhua Hospital, Affiliated to Shanghai Jiao Tong University, School of Medicine, No. 1665 Kongjiang Road, Shanghai 200092, China; 3 Department of General Surgery, Changshu Hospital, Affiliated to Suzhou University, No.1 Shuyuan Road, Changshu 215500, China

**Keywords:** Oridonin, Gallbladder cancer, Apoptosis, Cell cycle arrest, Mitochondrial pathway

## Abstract

**Background:**

Gallbladder cancer is the most frequent malignancy of the bile duct with high aggressive and extremely poor prognosis. The main objective of the paper was to investigate the inhibitory effects of oridonin, a diterpenoid isolated from *Rabdosia rubescens*, on gallbladder cancer both *in vitro* and *in vivo* and to explore the mechanisms underlying oridonin-induced apoptosis and cell cycle arrest.

**Methods:**

The anti-tumor activity of oridonin on SGC996 and NOZ cells was assessed by the MTT and colony forming assays. Cell cycle changes were detected by flow cytometric analysis. Apoptosis was detected by annexin V/PI double-staining and Hoechst 33342 staining assays. Loss of mitochondrial membrane potential was observed by Rhodamine 123 staining. The *in vivo* efficacy of oridonin was evaluated using a NOZ xenograft model in athymic nude mice. The expression of cell cycle- and apoptosis-related proteins *in vitro* and *in vivo* was analyzed by western blot analysis. Activation of caspases (caspase-3, -8 and -9) was measured by caspases activity assay.

**Results:**

Oridonin induced potent growth inhibition, S-phase arrest, apoptosis, and colony-forming inhibition in SGC996 and NOZ cells in a dose-dependent manner. Intraperitoneal injection of oridonin (5, 10, or 15 mg/kg) for 3 weeks significantly inhibited the growth of NOZ xenografts in athymic nude mice. We demonstrated that oridonin regulated cell cycle-related proteins in response to S-phase arrest by western blot analysis. In contrast, we observed inhibition of NF-κB nuclear translocation and an increase Bax/Bcl-2 ratio accompanied by activated caspase-3, caspase-9 and PARP-1 cleavage after treatment with oridonin, which indicate that the mitochondrial pathway is involved in oridonin-mediated apoptosis.

**Conclusions:**

Oridonin possesses potent anti-gallbladder cancer activities that correlate with regulation of the mitochondrial pathway, which is critical for apoptosis and S-phase arrest. Therefore, oridonin has potential as a novel anti-tumor therapy for the treatment of gallbladder cancer.

## Background

Gallbladder cancer, the most frequent malignancy of the bile duct, is a highly aggressive, lethal neoplasm that is associated with high mortality and extremely poor prognosis [1,2]. Despite recent advances in diagnostic and therapeutic approaches, the 5-year survival rate is generally 13%–30% [3]. Because of the absence of specific symptoms and signs, it is usually detected at an advanced stage [4]. Surgical resection is the only potentially curative therapy for gallbladder cancer [5]. Moreover, the majority of patients have frequent recurrences following surgery and unsatisfactory results after chemotherapy or radiotherapy [6]. Therefore, novel effective therapeutic drugs are urgently needed for this deadly disease.

**Figure 1:**
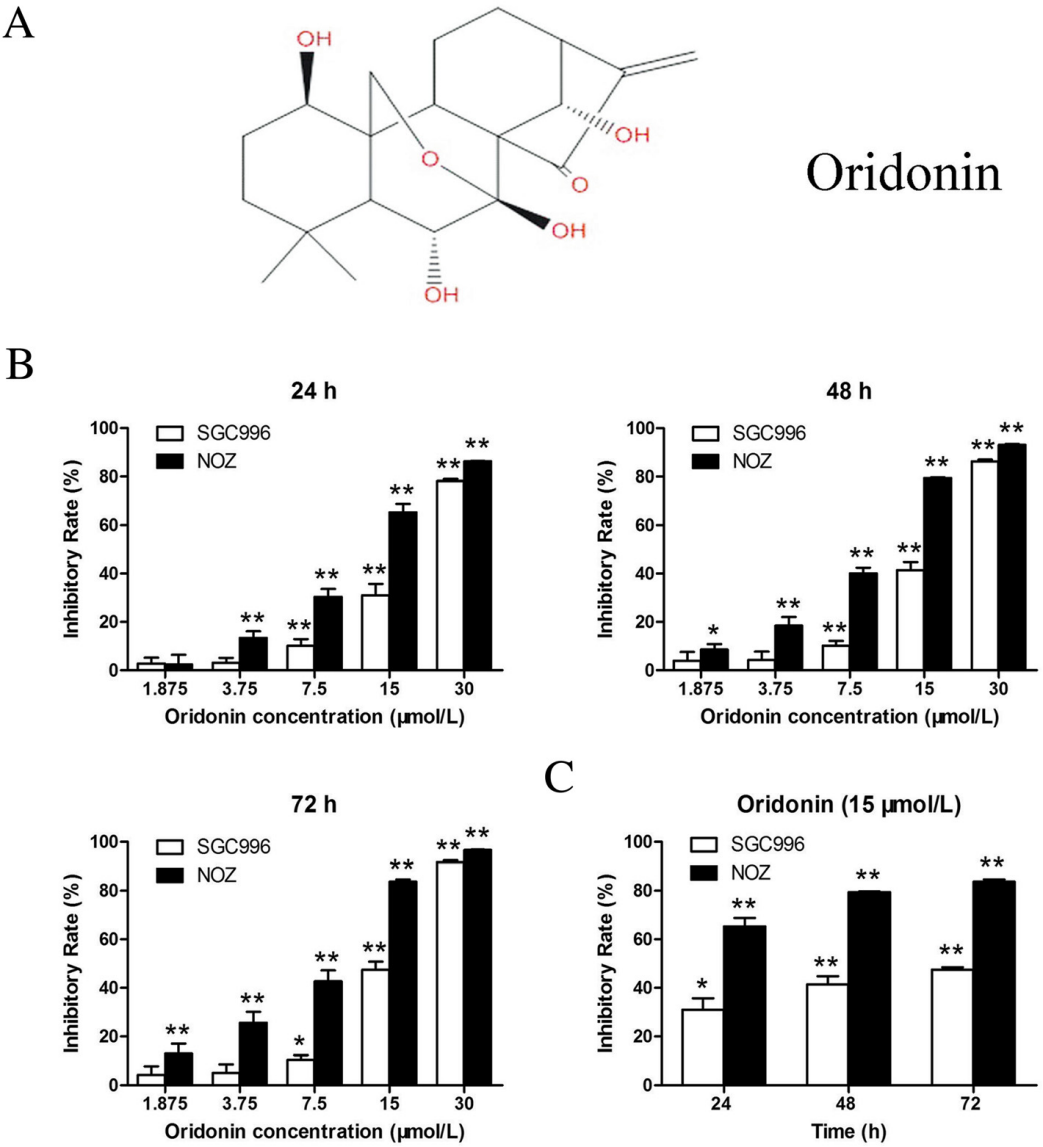
**Oridonin inhibits the proliferation of gallbladder cancer cells. (A)** Chemical structure of oridonin. **(B)** SGC996 and NOZ cells were treated with various concentrations of oridonin or vehicle for 24, 48, and 72 h. **(C)** Cells were incubated with 15 μmol/L oridonin for various time periods. Effects of oridonin on cell proliferation were determined by the MTT assay. Data are expressed as the mean ± SD (n = 5). The data were obtained from 3 independent experiments. *P < 0.05, **P < 0.01 vs. the control group.

Oridonin (Figure 1A), an active diterpenoid compound purified from the Chinese herb *Rabdosia rubescens*, has been reported to have various pharmacological and physiological effects, such as anti-inflammatory, anti-bacterial, and even anti-tumor effects [7]. Studies have shown that oridonin induces apoptosis in cells derived from a variety of cancers, including breast cancer, hepatocellular carcinoma, colorectal cancer, gastric cancer, and pancreatic cancer [8-12]. However, ordonin has very little effect on normal human cells such as lymphoid cells and fibroblasts [13,14]. In recent years, oridonin has attracted more attention because it stimulates cell cycle arrest and apoptosis in a wide variety of tumors both *in vivo* and *in vitro*. Numerous proteins and pathways have been shown to regulate oridonin-mediated apoptosis, including the cysteine-dependent aspartate-specific proteases (caspase) family, the Bcl-2 family, the mitogen-activated protein kinase (MAPK) family, the nuclear factor-kappaB (NF-κB), p53, and phosphoinositide 3-kinase (PI3K) signal transduction pathways [12,13,15,16]. However, systematic studies on how oridonin affects gallbladder cancer have not been reported.

In this study, we investigated whether oridonin induced growth inhibition, cell cycle arrest in gallbladder cancer cells *in vitro* and *in vivo*, and we explored the possible mechanisms of action, which could provide experimental evidence for the potential application of oridonin as a new natural anti-tumor medicine for gallbladder cancer.

## Methods

### Materials

Oridonin was purchased from Sigma-Aldrich (St. Louis, MO, USA). For *in vitro* studies, oridonin was dissolved in dimethyl sulfoxide (DMSO) to create a stock solution (0.1 mol/L), which was stored at −20°C. To prepare working solutions, the stock solution was further diluted with culture media to yield the desired oridonin concentration. Control cells were treated with an equal volume of vehicle. The DMSO concentration was kept below 0.1% in cell culture and did not have any detectable effect on cell growth or cell death.

3-[4,5-dimethylthiazol-2-yl]-2,5-diphenyl-tetrazolium bromide (MTT), Hoechst 33342, annexin V-FITC, propidium iodide (PI), and Rhodamine 123 were purchased from Sigma Chemical Co. (St. Louis, MO, USA). Primary antibodies against caspase-3, caspase-9, NF-κB, Bax, Bcl-2, PARP-1, cytochrome *c*, β-actin, and secondary antibodies (goat-anti-rabbit or goat-anti-mouse) were purchased from Cell Signaling Technology (Danvers, MA, USA). Antibodies against cyclin A, cyclin B1, and cyclin D1 were purchased from Epitomics (Burlingame, CA, USA).

### Cell lines and cell culture

The human gallbladder cancer cell lines SGC996 and NOZ were purchased from the Cell Bank of Type Culture Collection of Chinese Academy of Sciences (Shanghai, China). SGC996 cells were cultured in Rosewell Park Memorial Institute (RPMI) 1640 medium (Gibco, USA). NOZ cells were cultured in William’s medium (Gibco). The media for both cell lines were supplemented with 10% fetal bovine serum (Gibco), 100 μg/mL streptomycin, and 100 U/mL penicillin (Hyclone, USA) and maintained at 37°C in a humidified atmosphere with 5% CO_2_.

### Cell viability assay

The viability of cells treated with oridonin was measured by the MTT assay. During the logarithmic growth phase, cells were collected and seeded in 96-well plates at a density of 5 × 10^3^ cells/well and cultured. After 12 h of incubation, the cells were treated with oridonin (0, 1.875, 3.75, 7.5, 15, and 30 μmol/L) for 24, 48, and 72 h. After treatment, 20 μL of MTT solution (5 mg/mL) was added to each well and the cells were then incubated at 37°C for 4 h. The culture medium was then replaced with 100 μL of DMSO. The absorbance of the solution at 490 nm was measured with a microplate reader (Bio-Tek, USA). The results represent the average of 5 parallel samples. The cell inhibitory rate was calculated as follows: Inhibitory rate (%) = (A_490 control_ – A_490 sample_)/(A_490 control_ – A_490 blank_) × 100%.

### Colony forming assay

SGC996 and NOZ cells were plated into a 6-well culture plate (500 cells/well) and allowed to adhere for 10 h before treatment. After adherence, cells were treated with oridonin (0.75, 1.5, and 3 μmol/L). After 48 h, the oridonin-containing medium was removed, and the cells were allowed to form colonies in complete medium for 14 days. Then, the colonies were fixed with a solution of acetic acid and methanol (1:3) for 15 min, stained with 5% Giemsa (Sigma-Aldrich) for 30 min, and counted manually. Digital images were taken of stained single clones observed under a microscope (Leica, Germany).

### Cell cycle analysis by flow cytometry

Cells were treated with oridonin (7.5, 15, and 30 μmol/L) for 48 h. Both floating and adherent cells were collected and washed with cold phosphate buffered saline (PBS) and fixed with 70% ethanol overnight at 4°C. Cells were then treated with staining buffer (PBS containing 1 mg/mL PI and 10 mg/mL RNase A; Sigma-Aldrich) at 37°C in the dark for 30 min. The samples were analyzed with a flow cytometer (BD, San Diego, CA, USA).

### Annexin V/PI staining assay for apoptosis

The cells were treated with oridonin (7.5, 15, and 30 μmol/L) for 48 h. After washing twice with cold PBS, the cells were resuspended at a density of 1 × 10^6^ cells/mL. Then, 100 μL of binding buffer containing 2.5 μL of annexin V-FITC and 1 μL of 100 μg/mL PI was added to these cells and incubated for 30 min in the dark. Finally, the samples were analyzed by a flow cytometer (BD).

### Observation of morphological changes with Hoechst 33342 staining

After treatment with oridonin (7.5, 15, and 30 μmol/L) for 48 h, the cells were washed in PBS and fixed with methanol:acetic acid (3:1) at room temperature for 15 min. Then, the cells were washed in PBS and stained with 5 μg/mL Hoechst 33342 for 10 min at 37°C. Finally, the cells were washed with PBS and observed under a fluorescence microscope (Leica, Germany).

### Detection of mitochondrial membrane potential (ΔΨm) variation by flow cytometry

ΔΨm was analyzed by flow cytometry following Rhodamine 123 staining [17]. After treatment with oridonin (7.5, 15, and 30 μmol/L) for 48 h, the culture medium was removed and the cells were washed with PBS twice and then stained in Rhodamine 123 staining solution (5 μg/mL) at 37°C for 20–30 min. The samples were analyzed by using a flow cytometer (BD).

### Western blot analysis

The cells were incubated with oridonin (7.5, 15, 30 μmol/L) for 48 h, and then the adherent and floating cells were harvested, washed twice with ice-cold PBS, and lysed in RIPA buffer (Beyotime Institute of Biotechnology, Beijing, China) and protease inhibitor (Roche Applied Science, Indianapolis, IN, USA) at 4°C for 5 min. After centrifugation at 14,000× *g* for 5 min, the protein content of the supernatant was determined by the bicinchoninic acid (BCA) assay kit (Beyotime) according to the manufacturer’s instructions. The protein lysates (40 μg/lane) were separated by 10% SDS-PAGE and blotted onto nitrocellulose membranes (Millipore, Bedford, MA, USA). Each membrane was blocked with 5% skim milk, and then incubated with the indicated primary antibodies against caspase-3, caspase-9, NF-κB, Bax, Bcl-2, PARP-1, cytochrome *c*, cyclin A, cyclin B1, cyclin D1, and β-actin overnight at 4°C. Subsequently, the membrane was incubated with the secondary antibodies (HRP-conjugated goat anti-rabbit or goat anti-mouse IgG) for 1 h at room temperature and the formed immunocomplex was visualized by using a Gel Doc 2000 (BioRad, USA). The mitochondrial and cytosol fractions were extracted using the mitochondria extraction kit (Beyotime).

### Caspases activity assay

Cells (5 × 10^5^/dish) were seeded in 10 cm dishes and treated with 7.5, 15 and 30 μmol/L of oridonn for 48 h. After different treatments, cells were collected, washed three times with PBS and resuspended in Cell lysates buffer (25 mM Tris –HCl, pH 7.5, 20 mM MgCl2, and 150 mM NaCl, 1% Triton X-100, 25 μg/ml leupeptin, and 25 μg/ml aprotinin) for 15 min on ice. Lysates were centrifuged at 16,000 × g for 15 min, the supernatants collected and protein concentration determined by Bradford Protein Assay Kit (Beyotime, China). Cellular extracts (30 μg) were then incubated in a 96-well microtitre plate with 20 ng Ac-DEVD-pNA, Ac-IETD-pNA and Ac-LEHD-pNA (Beyotime) for 2 h at 37°C. Caspases activity was measured by cleavage of the Ac-DEVD-pNA, Ac-IETD-pNA and Ac-LEHD-pNA substrate to pNA, the absorbance of which was measured at 405 nm. Relative caspase activity was calculated as a ratio of emission of treated cells to untreated cells.

### Experimental animals

Male athymic nude mice (4–6 weeks old with an initial body weight of 20 ± 2 g) were obtained from Shanghai SLAC Laboratory Animal Co., Ltd. (Shanghai, China). The animals were acclimatized at a temperature of 25°C ± 2°C and a relative humidity of 70% ± 5% under natural light/dark conditions for 1 week with *ad libitum* access food and water. All animal treatments were performed in strict accordance with international ethical guidelines and the National Institutes of Health Guide for the Care and Use of Laboratory Animals. The animal experiments were approved by the Institutional Animal Care and Use Committee of Shanghai Jiao Tong University.

### *In vivo* tumor xenograft study

NOZ cells in log-phase growth were resuspended in serum-free culture medium (at a density of 1 × 10^6^ cells in 0.2 mL), and then tumor xenografts were established by subcutaneous inoculation of these NOZ cells into the right flank of nude mice. Twenty-four hours post-inoculation, the mice were randomly divided into 4 groups (10 mice/group). One group was administered vehicle (10% DMSO and 90% PBS) intraperitoneally (IP) and the others were administered oridonin (5, 10, and 15 mg/kg) IP in a volume of 0.2 mL every 2 days for up to 20 days. Tumor volume was measured using calipers and estimated according to the following formula: tumor volume (mm^3^) = (L × W^2^)/2, where L and W represent the length and width of the tumor, respectively. On day 21, the animals were sacrificed, and the tumor tissue was removed and weighed. Xenograft tumors in control mice and in mice treated with 15 mg/kg oridonin were harvested and cut into sections for western blot analysis.

### Western blot analysis of tumor tissues

Protein was routinely extracted from tumor tissues using RIPA buffer. Protein concentration was measured using a BCA assay kit (Beyotime). Tumor tissue extracts containing 80 μg of protein were separated by 10% SDS-PAGE, and then the resolved proteins were transferred to nitrocellulose membranes. After blocking with 5% skim milk, the membranes were incubated with individual primary antibodies overnight at 4°C, and the bound antibodies were detected with an HRP-conjugated goat anti-rabbit or goat anti-mouse IgG for 1 h. The formed immunocomplexes were visualized by using the Gel Doc 2000.

### Statistical analysis

All data and results were confirmed in at least 3 independent experiments. Data are expressed as the means ± SD. Differences between 2 sample means were assessed by Student’s *t*-test using SPSS v19.0 software (IBM Corporation). A *P* value of less 0.05 was considered statistically significant.

## Results

### Oridonin inhibits the proliferation of gallbladder cancer cells

**Figure 2:**
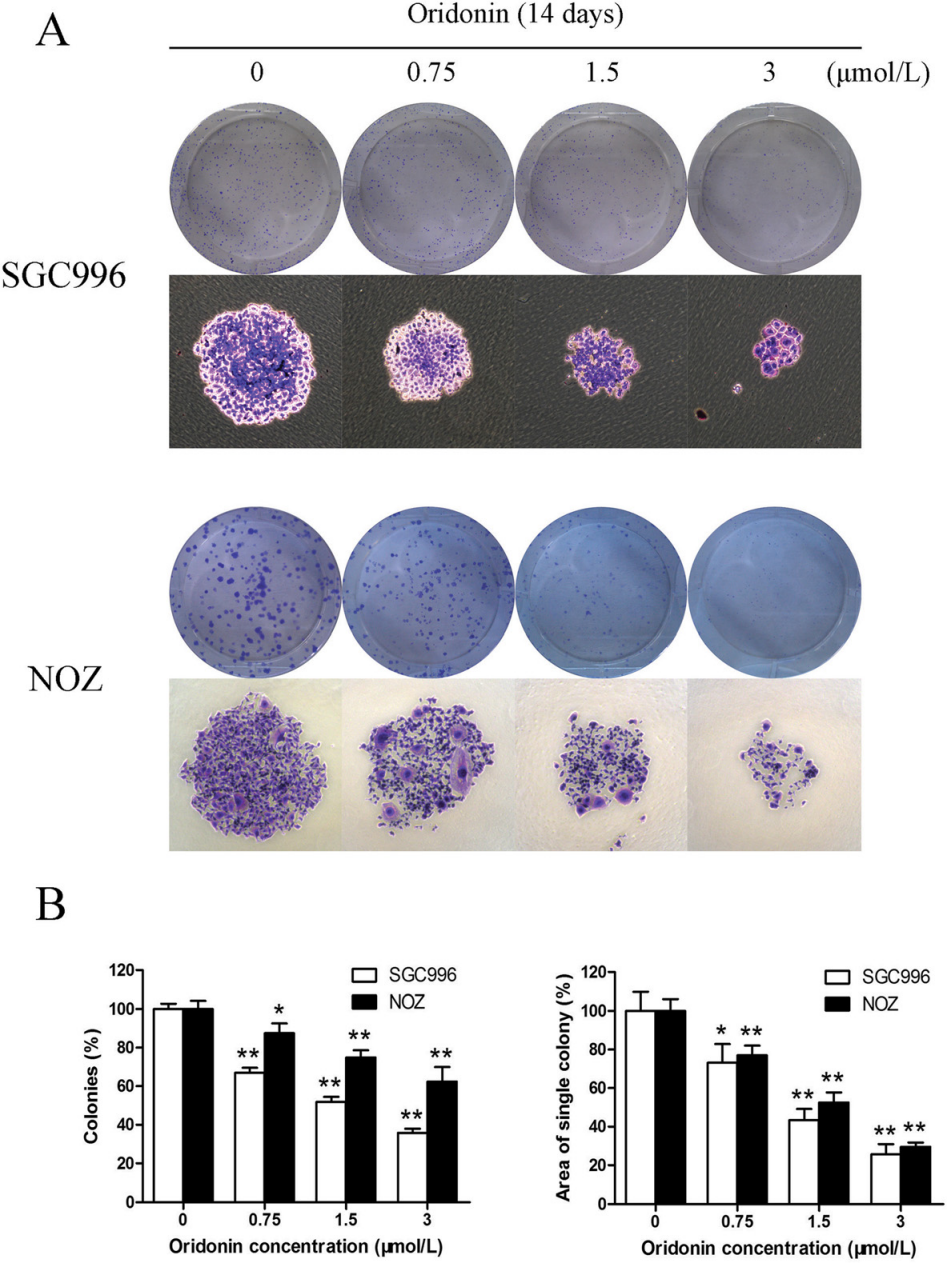
**Oridonin suppresses the colony forming ability of gallbladder cancer cells.** SGC996 and NOZ cells were treated with oridonin (0.75, 1.5, and 3 μmol/L) and were allowed to form colonies in fresh medium for 14 days. The photomicrographic differences **(A)** and influence of colonies (mean ± SD, n = 3) **(B)** on colony formation are shown. All data were from 3 independent experiments. *P < 0.05, **P < 0.01 vs. the control group.

To investigate the effect of oridonin on the proliferation of cells, SGC996 and NOZ cells were treated with various concentrations of oridonin and cell proliferation was detected by the MTT assay. Oridonin exhibited a potent cytotoxic effect on SGC996 and NOZ cells in a time- and dose-dependent manner (Figure 1). These 2 tumor cell lines showed different sensitivity to oridonin. NOZ cells were more sensitive to oridonin than SGC996 cells. At 30 μmol/L oridonin, the growth of SGC996 and NOZ cells was almost completely inhibited. The ability of gallbladder cancer cells to form colonies in the presence of oridonin was assessed by the flat plate colony forming assay (Figure 2A). The assay results showed that oridonin induced a dose-dependent decrease in colony formation. Moreover, statistical analysis demonstrated that the mean sizes of the colonies in the control were larger than those in the oridonin-treated group (Figure 2B). The results indicate that oridonin may have a long-term effect on the proliferation of gallbladder cancer cells.

### Oridonin induces cell cycle arrest at S-phase in gallbladder cancer cells

**Figure 3:**
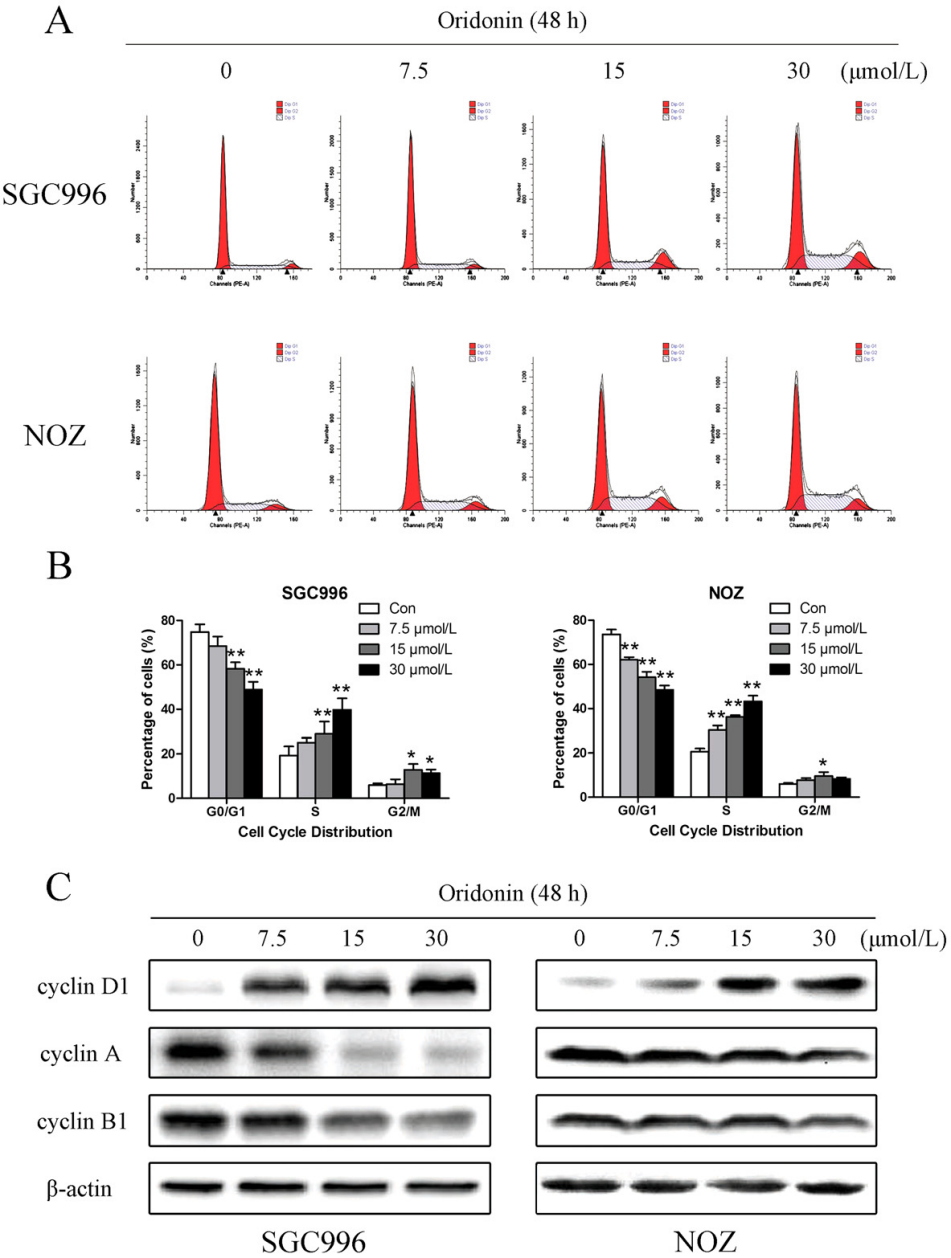
**Oridonin induces cell cycle arrest at S-phase and regulates the expression of cell cycle-related proteins in gallbladder cancer cells.** SGC996 and NOZ cells were treated with oridonin (7.5, 15, and 30 μmol/L) for 48 h. **(A)** The cell cycle phases of the treated cells were evaluated by flow cytometry. **(B)** Data were expressed as mean ± SD (n = 3). **(C)** The expression levels of cyclin A, cyclin B1, and cyclin D1 were measured by western blot analysis, and β-actin was used as a loading control. Results were representative of 3 independent experiments. *P < 0.05, **P < 0.01 vs. the control group.

To determine whether the effects of oridonin on the proliferation of gallbladder cancer cells are mediated by inhibition of cell cycle progression, the cell cycle phases of treated cells were analyzed by flow cytometry. Oridonin-mediated changes in the cell cycle of SGC996 and NOZ cells are shown in Figure 3A. After treatment with oridonin for 48 h, the percentage of G0/G1-phase cells dramatically decreased (from 74.86% to 48.90% for SGC996 cells and from 53.80% to 23.91% for NOZ cells), whereas the percentage of cells in S-phase dramatically increased (from 19.22% to 39.80% for SGC996 cells and from 35.76% to 74.02% for NOZ cells) compared to control cells. Higher oridonin concentrations had additional effects on the distribution of gallbladder cancer cells in the cell cycle. These results indicate that oridonin induced S-phase arrest in a dose-dependent manner.

We also assessed the levels of the G0/G1-related protein cyclin D1, the S-related protein cyclin A, and the G2/M-related protein cyclin B1 by western blot analysis in SGC996 and NOZ cells (Figure 3C). Oridonin treatment for 48 h down-regulated cyclin A and cyclin B1 and up-regulated cyclin D1, indicating that oridonin induces S-phase arrest in these cells.

### Oridonin induces apoptosis of gallbladder cancer cells

**Figure 4:**
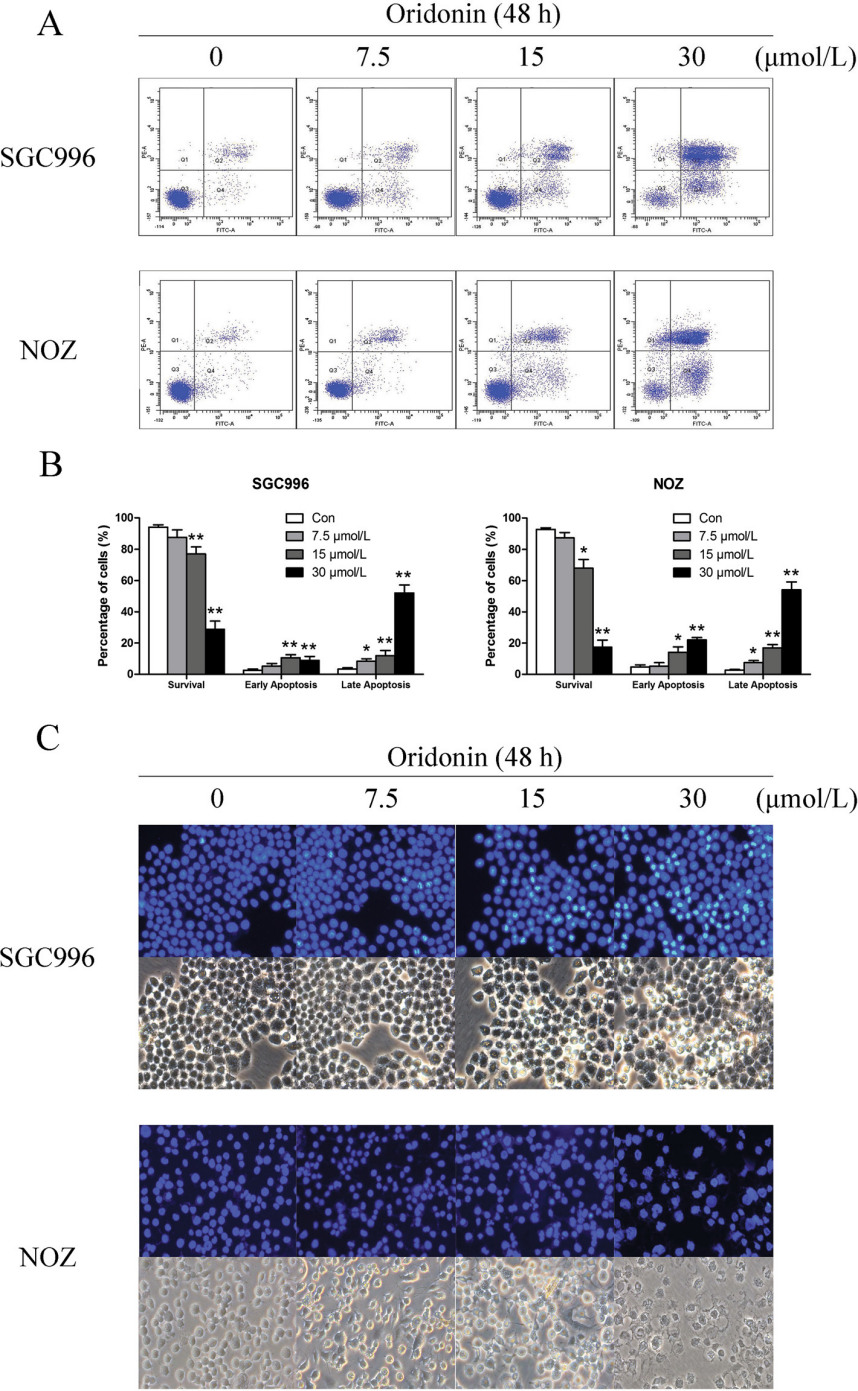
**Oridonin induces apoptosis in gallbladder cancer cells.** SGC996 and NOZ cells were treated with oridonin (7.5, 15, and 30 μmol/L) for 48 h. **(A)** Oridonin-treated SGC996 and NOZ cells were stained with annexin V-FITC/PI and analyzed by flow cytometry. **(B)** The percentage of apoptotic cells is presented as the mean ± SD (n = 3). **(C)** Changes in apoptotic nuclear morphology were observed by Hoechst 33342 staining and visualized by fluorescent microscopy. Results shown were representative data from 3 independent experiments. *P < 0.05, **P < 0.01 vs. the control group.

To assess the mechanism underlying oridonin-mediated growth inhibition, oridonin-treated SGC996 and NOZ cells were stained with annexin V-FITC and PI for flow cytometric analysis. Externalization of phosphatidylserine (PS) from the inner leaflet to the outer leaflet of the plasma membrane is a distinct phenomenon of early apoptosis. As annexin V possesses high affinity for PS, apoptotic cells can easily be detected by fluorescently labeling annexin V. In contrast, PI can detect necrotic cells due to its ability to permeate damaged cell membranes. Oridonin treatment induced a dose-dependent increase in both early and late stage apoptosis of SGC996 and NOZ cells (Figure 4A). Oridonin at a concentration of 30 μmol/L had a more significant apoptosis-inducing effect when compared to the number of apoptotic cells in the control group.

Apoptosis was also confirmed by examining the nuclear morphology by Hoechst 33342 staining. The cells in the control group were round and homogeneously stained, whereas oridonin-treated cells showed obvious chromatin condensation and fragmentation (Figure 4C). Moreover, the numbers of apoptotic nuclei containing condensed chromatin increased significantly as the oridonin concentration increased. Based on these morphologic changes, oridonin appeared to cause apoptosis of gallbladder cancer cells.

**Figure 5:**
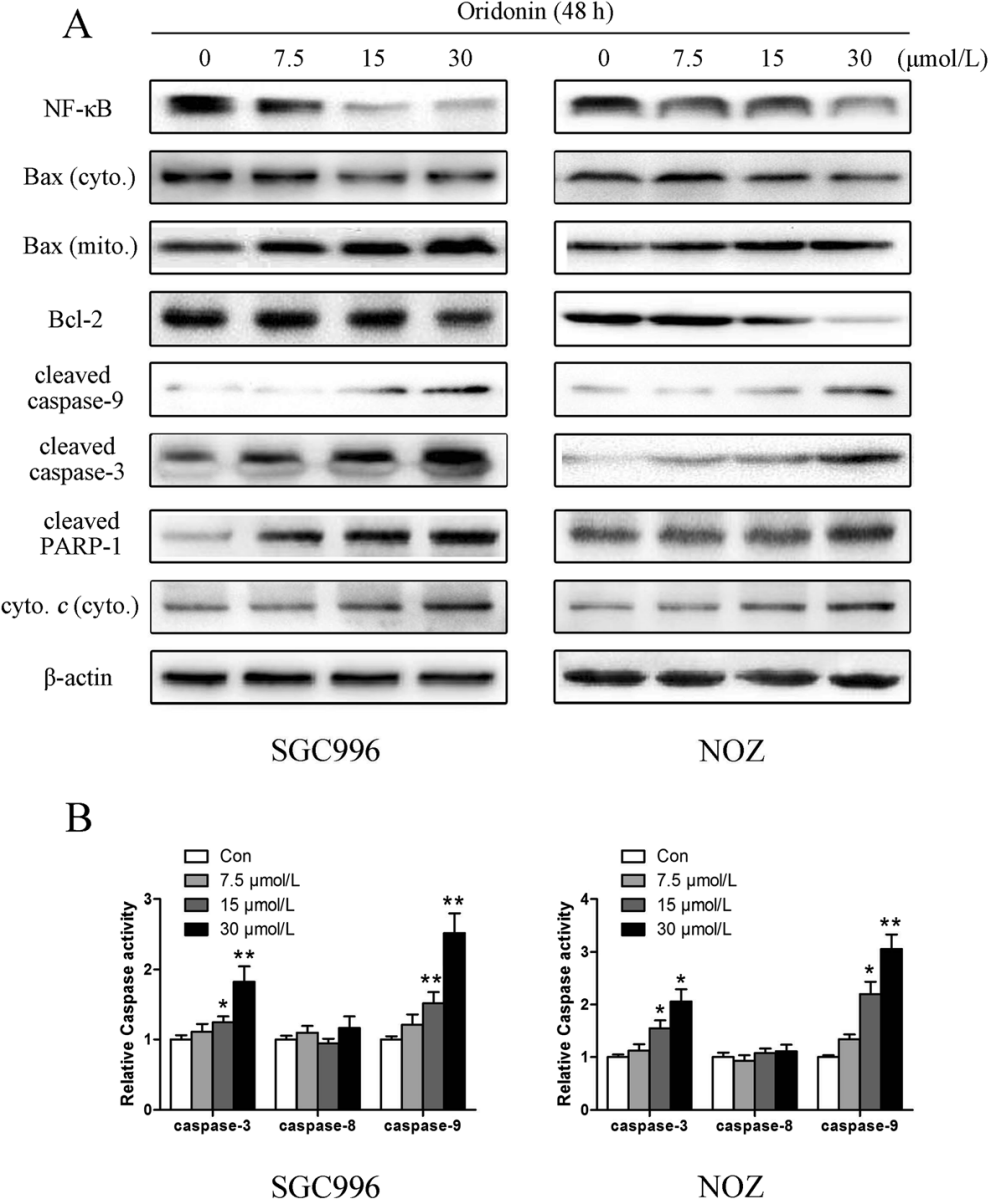
**Oridonin regulates the expression of apoptosis related proteins and caspases activities in gallbladder cancer cells.** SGC996 and NOZ cells were treated with oridonin (7.5, 15, and 30 μmol/L) for 48 h. **(A)** The expression levels of NF-κB, Bax, Bcl-2, cleaved caspase-9, caspase-3, cleaved PARP-1, cytochrome *c* were detected by western blot analysis, and β-actin was used as a loading control. **(B)** The data of the caspases activities were expressed as mean ± SD. The results shown were representative data from 3 independent experiments. *P < 0.05, **P < 0.01 vs. the control group.

It is well known that proteins in the caspase family, Bcl-2 family, NF-κB, and PARP play critical roles in the apoptotic process [18-21]. We assessed these apoptosis-related proteins by western blot analysis. Treatment with oridonin down-regulated Bcl-2, NF-κB, and up-regulated cleaved caspase-3, caspase-9, cleaved PARP-1, mitochondrial Bax and cytosol cytochrome *c* in a dose-dependent manner, which may be responsible, at least in part, for the apoptotic tendency of oridonin-treated SGC996 and NOZ cells (Figure 5A).

### Oridonin regulates caspase-3, -8 and -9 activation

The process of apoptosis involves a cascade of proteolytic activity, much of it carried out by caspases. To further characterize the apoptotic pathway activated by oridonin, we determined the kinetics of caspase activation. The activation of caspases in ordoin-treated cells was assessed using colorigenic tetrapeptide substrates, Ac-DEVD-pNA, AcIETD-pNA and Ac-LEHD-pNA, which have been shown to be selective for caspase-3, -8 and caspase-9-like enzymatic activities, respectively. Caspase-3, -8 and -9 activity induced by oridonin at 0, 7.5, 15 and 30 μmol/L after 48 h were measured using caspase-3, -8, -9 activity kit. oridonin induced the activity of both caspase-3 and -9 in SGC996 and NOZ cells in a dose-dependent manner (Figure 5B). But the activity of caspase-8 had no obvious changed which manifest that the signal pathway is independent of caspase-8 regulation.

### Oridonin induces disruption of mitochondrial integrity in gallbladder cancer cells

**Figure 6:**
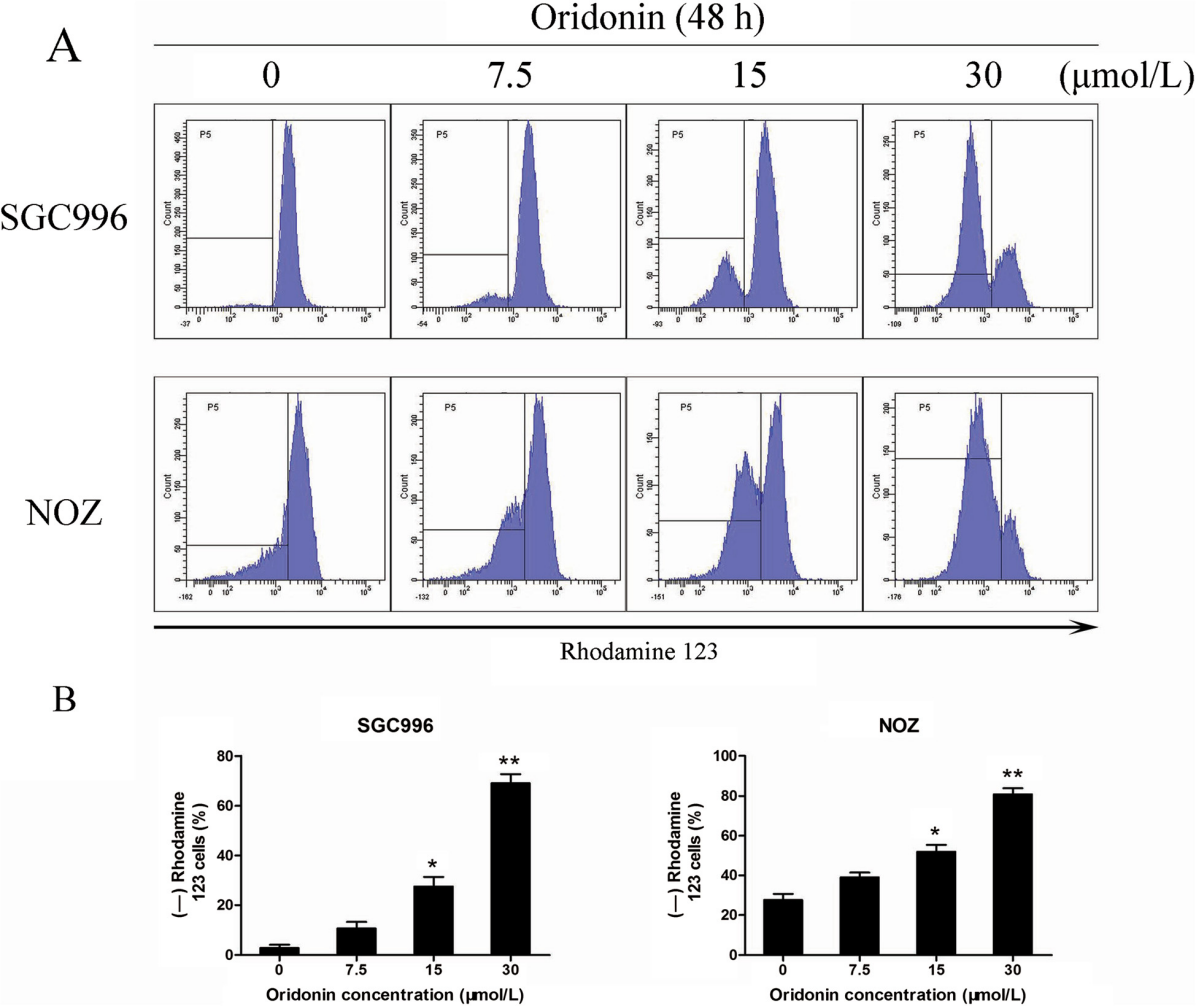
**Oridonin disrupts mitochondrial integrity in gallbladder cancer cells.** SGC996 and NOZ cells were treated with oridonin (7.5, 15, and 30 μmol/L) for 48 h. **(A)** Rhodamine retention was measured by flow cytometry. **(B)** The corresponding linear diagram shows the percentages of Rhodamine 123-negative cells and is expressed as the mean ± SD (n = 3). The results shown were representative data from 3 independent experiments. *P < 0.05, **P < 0.01 vs. the control group.

To investigate mitochondrial membrane potential (ΔΨm) changes induced by oridonin treatment, cells were stained with Rhodamine 123 and staining was detected by flow cytometry. The loss of the ΔΨm was reflected by a decrease in the intensity of Rhodamine 123 fluorescent staining, which was used to detect mitochondrial membrane integrity. Compared with the control cells, oridonin treatment increased the ratio of Rhodamine 123-negative cells from 2.85% to 69.1% for SGC996 cells and from 27.6% to 80.8% for NOZ cells in a dose-dependent manner (Figure 6). These findings suggest that oridonin could reduce mitochondrial membrane potential and induce mitochondrial dysfunction in gallbladder cancer cells.

### Oridonin represses the growth of NOZ cells *in vivo*

**Figure 7:**
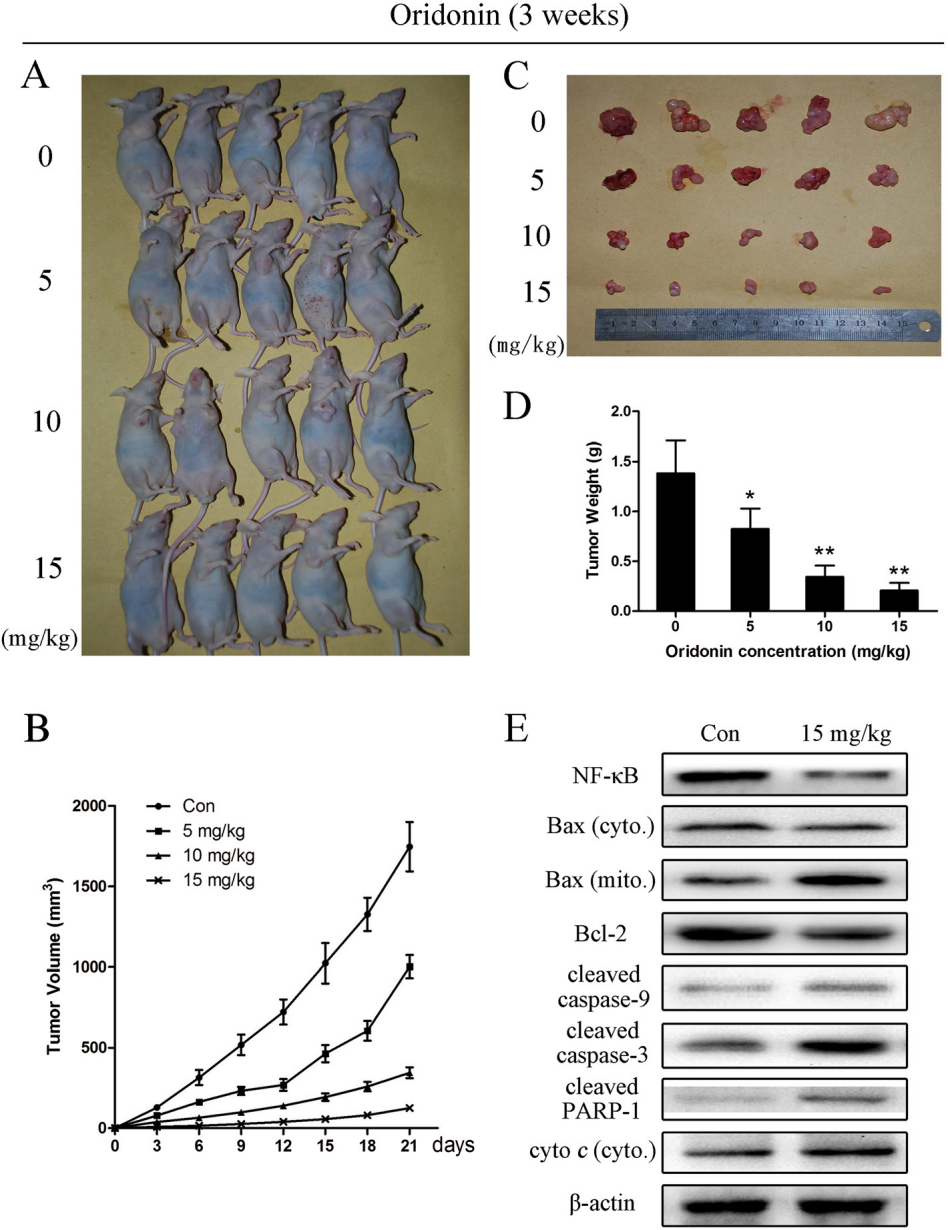
**Oridonin represses the growth of NOZ cells in athymic nude mice.** NOZ cells were subcutaneously injected into the right flank of the nude mice. The mice were then administered 0.2 mL of vehicle (10% DMSO and 90% PBS) or oridonin (5, 10, and 15 mg/kg) intraperitoneally every 2 days for up to 3 weeks. **(A)** Photos of 5 representative mice (n = 10) from each group were presented to show the sizes of the resulting tumors. **(B)** Tumor dimensions were periodically measured using calipers. **(C and D)** Tumors were excised from the animals and weighed. The data are expressed as the mean ± SD (n = 10). *P < 0.05, **P < 0.01 vs. the control group. **(E)** The expression of apoptosis-related proteins in tumor tissue was also assessed by western blot analysis, and β-actin was used as a loading control. The results shown were representative data from 3 independent experiments.

To determine the antitumor effect of oridonin *in vivo*, mice bearing NOZ cell tumors were administered oridonin or vehicle (10% DMSO and 90% PBS) every 2 days for up to 20 days. The tumors removed from these animals are shown in Figure 7A and 7C, and their mean weights and volumes are provided in Figure 7B and 7D. There was a marked reduction in tumor volume and tumor weight in mice treated with oridonin compared with control mice, and this reduction was dose dependent (Figure 7). The appearance of the tumors was in agreement with the statistical analysis of tumor volume data, which showed that oridonin treatment significantly inhibited tumor growth.

To determine whether the impact of oridonin on tumor growth inhibition was related to caspase-3, caspase-9, NF-κB, Bax, Bcl-2, PARP-1 and cytochrome *c*, we evaluated the levels of these apoptosis-related proteins in the gallbladder tumor tissues by western blot analysis. The results showed down-regulation of Bcl-2 and NF-κB and up-regulation of cleaved caspase-3, caspase-9, cleaved PARP-1, mitochondrial Bax and cytosol cytochrome *c* compared to the control group, which was in agreement with the results of the *in vitro* tests (Figure 7E).

## Discussion

Previous studies have shown that oridonin possesses anti‑proliferative and apoptotic activities against a variety cancer cells [22]. The results of this study demonstrated for the first time that oridonin decreased the viability of gallbladder cancer cell lines. The data from the MTT assays showed that oridonin inhibited the growth of SGC996 and NOZ cells in a time- and dose-dependent manner (Figure 1). Both the concentration and incubation time affected the cytotoxicity of oridonin. The colony forming assay showed similar results after a longer incubation period (Figure 2). The predominant mode of cell death in these cells was apoptosis, as determined by annexin V-FITC/PI staining (Figure 4A), characteristic changes in the morphology of Hoechst 33342-stained cells (Figure 4C), and cell cycle arrest studies (Figure 3A). Consistent with these *in vitro* results, treatment of NOZ xenografts in athymic nude mice with oridonin for 3 weeks significantly decreased the growth of the xenografts (Figure 7). These results provided strong evidence in support of the notion that oridonin has potent apoptotic effects against gallbladder cancer *in vitro* and *in vivo*.

It is well known that apoptosis is a programmed process that is essential for the development of most metazoans, and that deregulation of apoptosis is an indicator of cancer [23]. Generally, there are two major apoptosis pathways: the death-receptor-induced extrinsic pathway and the mitochondria-apoptosome-mediated apoptotic intrinsic pathway [24]. Mitochondria play an important role in regulating many cellular functions, and mitochondrial dysfunction has been proposed to be involved in many pathological processes [25]. In the present study, it is worth noting that there were similar levels of annexin-V-positive and Rhodamine 123-negative cells, which suggests that apoptosis is closely related to or dependent on the loss of ΔΨm (Figure 6).

In the mitochondrial pathway, NF-κB, a pro-survival transcription factor, controls the inflammatory and immune response as well as other genetic programs that are central to cell proliferation and cell survival and decrease the sensitivity of cancer cells to apoptosis [26]. NF-κB inhibits apoptosis by inhibiting Bcl-2 members and inhibitors of apoptosis [27]. In this study, inhibition of NF-κB nuclear translocation together with the down-regulation of its target Bcl-2 family member genes suggested that activation of NF-κB was inhibited by oridonin during tumor progression (Figure 5A).

The prime inducers of apoptotic pathways are pro-apoptotic and anti-apoptotic Bcl-2 family proteins and caspases. During apoptosis, the permeability of the mitochondrial membrane increased, leading to a loss of membrane potential and release of cytochrome *c* into the cytosol, which binds to apoptotic protease activating factor-1 (Apaf‑1) [28]. The Bcl-2 and Bcl-xL proteins have been identified as anti-apoptotic proteins, which bind to the outer membrane of the mitochondrion and prevent the release of cytochrome *c*[29]. The pro-apoptotic members of this family, Bax and Bak, are responsible for permeabilizing the membrane under stress and promoting the release of cytochrome *c* from the mitochondria [30,31]. It has been suggested that a high Bax to Bcl-2 ratio can cause ΔΨm collapse, release of cytochrome *c*, and subsequent apoptosis [32]. Our results show that oridonin significantly decreased Bcl-2 and induced the translocation of Bax to the mitochondria with the release of cytochrome *c* into the cytosol, suggesting that mitochondria are involved in oridonin-induced apoptosis (Figure 5A).

Caspases, a family of cysteases, play a crucial role in apoptosis progression, morphological changes, and DNA fragmentation [33]. Two distinct pathways of apoptosis have been identified as mitochondria-initiated apoptosis occurs through caspase-9; the death receptor-mediated pathway requires caspase-8 [34]. Bcl-2 inhibits the apoptotic process and promotes cell survival, and Bax acts within the mitochondria to induce the release of cytochrome *c*, leading to caspase-9 activation, and subsequent caspase-3 activation. Caspase-3 is one of the most important executioner caspases, and it is capable of cleaving many important cellular substrates such as PARP [35]. In our study, oridonin treatment activated caspase-3 and caspase-9, regulated the cleavage of PARP-1 (Figure 5A). Furthermore, ordonin raised the enzymatic activity of caspase-3 and caspase-9 significantly but not caspase-8 (Figure 5B), which suggested involvement of mitochondrial death pathways in oridonin-induced apoptosis. When we investigated the mechanisms by which oridonin manifests its effects against gallbladder cancer in an animal model, the results were in agreement with those of the *in vitro* tests (Figure 7E).

Progression through the various phases of the cell cycle is a tightly regulated process involving the various cyclins and cyclin-dependent kinases (Cdks), each of which function at different cell cycle phases [36]. The complex of cyclin A and Cdk2 initiates DNA synthesis and progression through S-phase [37]. As suggested by our cell cycle analysis data, oridonin arrested SGC996 and NOZ cells at S-phase (Figure 3A), which might be due to down-regulation of cyclin A and cyclin B1 and up-regulation of cyclin D1 (Figure 3C).

## Conclusions

In summary, our study showed that oridonin is a potent growth inhibitor of gallbladder cancer *in vitro* and *in vivo*. Growth inhibition was dose-dependent and was related to S-phase arrest. Oridonin also caused a marked increase in apoptosis, which was determined by characteristic morphological changes, increased numbers of apoptotic cells, and the loss of ΔΨm. Furthermore, inhibition of NF-κB nuclear translocation and an increased Bax/Bcl-2 ratio was mediated by activated caspase-3 and caspase-9 and PARP-1 cleavage. Taken together, these observations indicate that the mitochondrial pathway is involved in apoptosis induced by oridonin treatment. Oridonin has potential as a novel anti-tumor therapeutic strategy for the treatment of gallbladder cancer.

## Competing interests

The authors declare that they have no competing interests.

## Authors’ contributions

BRF and SYJ were responsible for the design of the experiments, execution of experiments, data statistics, and writing of the manuscript. LTY, JL and HYP participated in performing the experiments, and in the data analysis. TZJ helped in the study design. WH, DQ, CY, MJS, and DQC participated in discussion and data interpretation. WXS, LML, and WWG conceived of the study and revised the manuscript. LYB and GJF were responsible for the funding application, and supervision and management of the project. All authors have contributed to and approved the final manuscript.

## Pre-publication history

The pre-publication history for this paper can be accessed here:

http://www.biomedcentral.com/1471-2407/14/217/prepub
